# The Lothian Birth Cohort 1936: a study to examine influences on cognitive ageing from age 11 to age 70 and beyond

**DOI:** 10.1186/1471-2318-7-28

**Published:** 2007-12-05

**Authors:** Ian J Deary, Alan J Gow, Michelle D Taylor, Janie Corley, Caroline Brett, Valerie Wilson, Harry Campbell, Lawrence J Whalley, Peter M Visscher, David J Porteous, John M Starr

**Affiliations:** 1 Department of Psychology, University of Edinburgh, 7 George Square, Edinburgh EH8 9JZ, UK; 2 Scottish Council for Research in Education, University of Glasgow, Glasgow, UK; 3 Centre for Public Health and Primary Care Research, University of Edinburgh, Edinburgh, UK; 4 Department of Mental Health, University of Aberdeen, Aberdeen, UK; 5 Genetic Epidemiology, Queensland Institute of Medical Research, Brisbane, Australia; 6 Medical Genetics Section, Molecular Medicine Centre, University of Edinburgh, Edinburgh, UK; 7 Department of Geriatric Medicine, University of Edinburgh, Edinburgh, UK

## Abstract

**Background:**

Cognitive ageing is a major burden for society and a major influence in lowering people's independence and quality of life. It is the most feared aspect of ageing. There are large individual differences in age-related cognitive changes. Seeking the determinants of cognitive ageing is a research priority. A limitation of many studies is the lack of a sufficiently long period between cognitive assessments to examine determinants. Here, the aim is to examine influences on cognitive ageing between childhood and old age.

**Methods/Design:**

The study is designed as a follow-up cohort study. The participants comprise surviving members of the Scottish Mental Survey of 1947 (SMS1947; N = 70,805) who reside in the Edinburgh area (Lothian) of Scotland. The SMS1947 applied a valid test of general intelligence to all children born in 1936 and attending Scottish schools in June 1947. A total of 1091 participants make up the Lothian Birth Cohort 1936. They undertook: a medical interview and examination; physical fitness testing; extensive cognitive testing (reasoning, memory, speed of information processing, and executive function); personality, quality of life and other psycho-social questionnaires; and a food frequency questionnaire. They have taken the same mental ability test (the Moray House Test No. 12) at age 11 and age 70. They provided blood samples for DNA extraction and testing and other biomarker analyses. Here we describe the background and aims of the study, the recruitment procedures and details of numbers tested, and the details of all examinations.

**Discussion:**

The principal strength of this cohort is the rarely captured phenotype of lifetime cognitive change. There is additional rich information to examine the determinants of individual differences in this lifetime cognitive change. This protocol report is important in alerting other researchers to the data available in the cohort.

## Background

### Normal cognitive ageing and its determinants

There are increasing numbers of elders in the UK population, and the problems of ageing are deservedly attracting more research interest [1]. Cognitive decline is the single most feared aspect of growing old [2]. Identifying the cerebral basis for age-related cognitive decline is amongst the greatest challenges to improving the health of older people [3]. Age-related cognitive decline reduces quality of life, is an increasing burden to sufferers and their families, and places a massive financial load on society [4]. The spectrum of decline ranges from normal cognitive ageing, through Mild Cognitive Impairment, to the dementias [5]. The Lothian Birth Cohort 1936 study addresses the milder end of the spectrum. This is an especially important problem because it involves such large numbers of people compared with the dementias.

As humans grow older, some important cognitive functions deteriorate, even in the absence of dementia [6]. Abilities such as memory, reasoning, and spatial ability all decline, on average, with age [7]. Not everyone declines equally: some people show marked cognitive declines as they grow older, whereas others maintain cognitive skills relatively well in old age [7]. Normal cognitive ageing is a continuous trait. It is rare to be able to study this phenomenon, because there are few samples that have had cognitive testing performed more than once and with a sufficient amount of time between the measurements. We previously described the distribution of normal cognitive change across almost 70 years, from age 11 to age 79 [8]. Before interventions can be found to enhance the lives of older people, the determinants of individual differences in general and specific aspects of cognitive ageing must be discovered.

Discovering the determinants of non-pathological cognitive ageing has scientific and practical value: it will be informative about the mechanisms of cognition, and it can suggest interventions to promote successful cognitive ageing [9]. The major determinant of cognitive function in old age is cognitive function in youth [8,10]. It accounts for about 50% of the variance, leaving about half unaccounted for. Therefore, it is valuable first to assess cognition in youth – when cognitive assessment is unaffected by the processes of age and age-related illness – and then re-assess cognition in old age. The Lothian Birth Cohort 1936 is rare in having such data. Among the other determinants of normal cognitive ageing are genetic, medical, psychological, and social and lifestyle factors [3,11-15].

The following are examples of factors that have some evidence of an association with differences in normal cognitive ageing: smoking [16], physical fitness [17,18], personality [19,20], cardiovascular disease [20,21], social and intellectual engagement [15,22], diet [23,24], brain white matter hyperintensities [25] and brain white matter integrity [26]. Many of the effects are small, and not all are replicated. Some of these effects may in part be caused by shared genetic effects. Much of this type of information is obtained from studies which lack truly pre-morbid cognitive ability. Such studies are unable to test for reverse causality: the possibility that it is the lifelong trait of cognitive ability that brings about individual differences in the putative 'cause' of cognitive ageing.

### Genetic contributions to normal cognitive ageing

The Lothian Birth Cohort 1936 study will examine, among other factors, genetic contributions to individual differences in normal cognitive ageing [27,28]. Additive genetic influences contribute well over half of the variance to cognitive ability in adult humans, including in old age [29-33]. There is evidence of a genetic contribution to cognitive change within old age [34,35], but most studies suffer from a poor phenotype, with cognitive change being assessed across a small period of time.

In smaller, prior follow-up samples from the Scottish Mental Surveys of 1932 and 1947 we reported significant contributions to variance in normal cognitive ageing from variation in the following genes: *APOE *[12], *COMT *[36], *PRNP *[37], *DISC1 *[38], and *BDNF *[39]. *APOE *provided a clear example of a genetic polymorphism that, in the same sample was related to cognitive ability in old age but not in youth [12]. All of these analyses used cognitive ability measures in old age adjusted for mental ability at age 11 in the Scottish Mental Survey data. Because we had data on both childhood IQ and IQ in old age, we found some genes to be related to both, such as *NCSTN *[40], and *KL *[41]. Overall, there are, as yet, few replicated genetic associations with normal cognitive ageing.

### The importance of speed of information processing in normal cognitive ageing

There is a search for simpler psychological functions that can account for age-related changes in higher-level, more complex cognitive functions. Constructs such as information processing speed, working memory, and executive functions have been cast in this role. Each of these functions is examined in the Lothian Birth Cohort 1936. Speed of information processing is assessed in particular detail, and at different levels of description. This construct is often suggested as a possible mediator of age-related changes in other cognitive functions including some aspects of memory [42-44]. Processing speed measures applied to human subjects range from psychometric-behavioural type tests (e.g. the Digit Symbol subtest of the Wechsler Adult Intelligence Scales), through cognitive-experimental assessments (e.g. various reaction time procedures), to psychophysical procedures (e.g. inspection time). In the Lothian Birth Cohort 1936 study information processing speed is operationalised into three levels. There are paper and pencil psychometric tests, and inspection time and reaction time measures.

Inspection time is a psychophysical measure of the efficiency of early visual processing. In meta-analyses inspection time correlates moderately highly with higher-level cognitive ability test scores, especially with those tests that are known to be sensitive to ageing [45]. It is sensitive to normal human ageing and there is evidence that it mediates some of the effect of age on scores on psychometric tests of higher cognitive functions [46]. It is slower in people with mild cognitive impairment [47] and dementia [48]. In genetic covariance analyses inspection time has some shared, additive genetic effects with higher mental test scores [49]. Inspection time will be used in the Lothian Birth Cohort 1936 as a sensitive, early indicator of more general cognitive decrements. Reaction time has also been conceived as a lower-level indicator of processing efficiency that can mediate effects of age on higher cognitive functions [42]. Reaction time has the additional advantage of providing measures of both speed and variability; both are sensitive to ageing from the 30s onwards [50]. In the present study the research team will be able to test whether molecular genetic influences on age-related changes in a number of key cognitive domains are accounted for by the same genes' influences on information processing variables such as reaction time and inspection time.

### The Scottish Mental Survey of 1947

The Lothian Birth Cohort 1936 comprises surviving participants of the Scottish Mental Survey 1947 (SMS1947) who now live in the Lothian area of Scotland. Most subjects resided in Edinburgh city. On June 4^th ^1947 almost all people born in 1936 and attending school in Scotland were tested on a valid cognitive ability test. The mental test was a version of the Moray House Test No. 12, which was concurrently validated against the Terman-Merrill revision of the Binet Scales [51]. There were 70,805 people tested out of a possible 75,211 people born in 1936 in the total population. SMS1947 survivors were at an interesting age to study cognitive ageing: mostly just under 70 when recruited into the Lothian Birth Cohort 1936. The childhood cognitive ability data provide a rarely-available baseline from which to calculate actual, almost life-long cognitive changes. The objectives of the study, listed below, are as stated in the successful application for programme grant funding from the UK charity Research Into Ageing.

### The objectives of the Lothian Birth Cohort 1936 study

1. To recruit and re-examine 1000 surviving participants of the Scottish Mental Survey 1947.

2. To collect detailed cognitive phenotypes, including assessments of major domains of cognitive function, and specialised assessments of speed of information processing.

3. To collect demographic, medical, and physiological data to relate to cognitive ageing differences.

4. To collect and store DNA and examine candidate genes for variation in normal cognitive ageing.

5. To test for genetic, medical, physiological, demographic and other determinants of individual differences in non-pathological cognitive ageing from age 11 to age 70.

6. To discover whether individual differences in cognition at age 70 are substantially accounted for by individual differences in speed of information processing.

7. To discover whether speed of information processing mediates the genetic influences on cognition at age 70.

8. To provide a high quality study that: may be continued as the participants grow older; and may be expanded to include, for example, brain imaging studies and further genetic studies.

9. To amass a uniquely valuable resource (stored phenotypes and DNA) that will be available to other researchers in human cognitive ageing. As candidate genes for cognitive ageing emerge in the future this resource will be a key test bed.

## Methods/Design

### Subjects and recruitment

Hereinafter, the Lothian Birth Cohort 1936 will be called the LBC1936. Ethics permission for the study protocol was obtained from the Multi-Centre Research Ethics Committee for Scotland (MREC/01/0/56) and from Lothian Research Ethics Committee (LREC/2003/2/29). The research was carried out in compliance with the Helsinki Declaration. All subjects gave written, informed consent. On behalf of the LBC1936 research team, and with the permission of, and under the management of the Director of Public Health for Lothian, the Lothian Health Board identified potential participants using the Community Health Index (CHI). This is a list of individuals in a given area who are registered with a general medical practitioner (GP). At any time, the CHI is not fully correct. Inaccuracies are due to the time necessary to update deaths or movements into and out of an area. The Lothian Health Board was requested by us to identify all individuals listed on the Lothian CHI who were born in 1936; that is, individuals who might have taken part in the Scottish Mental Survey of 1947. At the start of the study, 3810 people born in 1936 were identified on the Lothian CHI.

The initial contact with possible participants was made via Lothian Health Board, in a letter signed by the Director of Public Health. Researchers are not allowed to write to people on the CHI database directly. The mailing consisted of a letter from Lothian Health Board, an information sheet explaining why the contact was made by Lothian Health Board, an invitation letter written and signed by Professor Ian Deary (LBC1936 Study Director), an explanatory leaflet for the LBC1936 study, and a reply slip and a pre-paid reply envelope. The reply slip asked for normal contact details and date of birth, country of schooling, and schools attended. Individuals were also asked to indicate whether they were or were not interested in hearing more about the LBC1936 study. The reply slip was returned to the LBC1936 research team at the University of Edinburgh. This allowed the first direct contact between potential participants and researchers. Individuals were invited about 200–300 at a time (roughly according to postcode districts) to allow the LBC1936 study team to manage the responses efficiently.

**Figure 1:**
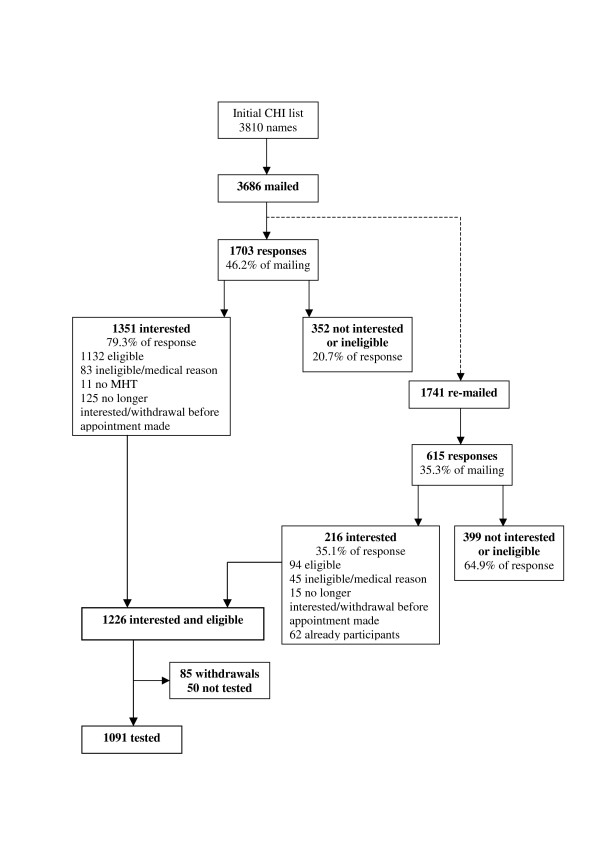
Recruitment flowchart for the Lothian Birth Cohort 1936.

Between June 2004 and November 2006, 3686 invitations were sent out in this way. This is lower than the original number of 1936-born individuals identified on the CHI because changes in circumstances of those listed can occur over a period of time. Of the 3686 individuals invited to participate, 1703 responded (46.2% of those contacted) (Figure 1). Two hundred and eighty six – or 16.8% of the respondents – were not interested in hearing more about the study. From the details included on the reply slip it was possible to determine that 209 of these refusals were from otherwise eligible individuals, 46 were ineligible, 9 were refusing for some medical reason, and 22 did not provide the details required to ascertain their eligibility. Sixty-six respondents did not indicate whether they were interested or not. However, 5 detailed medical conditions which would not allow them to participate, 2 were born in years other than 1936, and the remaining 59 were ineligible due to their country of schooling. 'Interested' responses were received from 1351 individuals (79.3% of the responses), although 83 of these were ineligible, 11 had not taken part in the Scottish Mental Survey of 1947, and a further 125 did not wish to participate after hearing more about the study. That left 1132 people who were both interested and eligible to take part in the LBC1936 study.

After 1900 individuals had been invited using the recruitment process detailed above, a second invitation was sent out to give the non-responders another chance to participate and to increase the response rate. Due to the confidentiality of the CHI, it was not possible to contact only those who had not responded to the first invitation. Rather, all individuals invited up to that time were re-mailed. In total, second invitations were sent to 1741 individuals (this is lower than the 1900 previously invited due to updating of the CHI over time). From this second invitation, 615 responses were received (35.3% of those mailed). Seventy eight were not marked as interested or not interested although, of these, 4 were returns from incorrect addresses, 52 noted that they had already participated, and 22 marked that they were ineligible. Of the 321 who responded as not interested, 44 were ineligible, and 5 had already attended; the remaining 272 gave no reason. Two hundred and sixteen individuals were interested, but this included 62 who were already participants in the study, and 45 who were ineligible. A further 15 did not wish to participate after hearing more about the study, leaving 94 who were both interested an eligible.

Having contacted all known 1936-born individuals living in Edinburgh and the surrounding area listed on the CHI (including the second letter to the first 1741 individuals invited), media advertisements were used to find any interested and eligible participants that may have been missed by this procedure. A call for volunteers was placed in a freely-distributed Edinburgh weekly paper in February 2007, and an advertisement was placed in an Edinburgh-based evening newspaper in March 2007. Due to the almost-blanket coverage of the CHI mailing, the response to the media advert was low, generating about 97 replies. These have been included in the numbers detailed above.

Combining both of the mailings and the media responses, 1226 individuals were interested and eligible for the study. This is, arguably, a good response from a cold mailing to a database that is imperfect and on which only a proportion will have been in education in Scotland in the relevant year. Eighty-five participants withdrew from the study before they were tested, and another 50 had not been contactable or were unable to attend an appointment before the end of testing in May 2007. In total, 1091 individuals became participants in the Lothian Birth Cohort 1936 study and completed the tests and questionnaires, detailed below (Figure 1).

### Participant Interview

All 1091 subjects were interviewed and tested individually by a trained psychologist and a research nurse at the Wellcome Trust Clinical Research Facility at the Western General Hospital, Edinburgh. The interview, which included cognitive, physical and other tests, took place in a single visit to the Facility. The assessments that each subject undertook are now described in the order in which they were administered. There was a break of at least 15 minutes for refreshments. According to staff availability, the position of the physical testing section was varied for some subjects.

#### Introduction to the LBC1936 study and consent

Participants were acquainted with the psychologist, reminded about the study, and given the opportunity to ask further questions. They gave written, informed consent to the study.

#### Social and medical history and medications

In an initial interview participants gave contact details, education (age at leaving school, further and higher education, and highest qualification obtained), main occupation (and that of their spouse for women), and age at retirement. Details of overcrowding at age 11 were obtained by asking about the number of rooms in their house at this time and the number of people living there. Smoking history and current alcohol consumption were noted. Disease history and current medications were obtained in a structured interview.

#### Hospital Anxiety Depression Scale

This assesses recent mood states [52]. It contains 7 items for anxiety and 7 for depression. The maximum score on each scale is 21, with probable anxiety or depression at scores of 11 or over.

#### Cognitive tests

##### Mini-Mental State Examination

This is commonly used as a screening test for dementia [53]. The maximum score is 30. Scores of less than 24 are used by some researchers and clinicians to indicate possible dementia.

##### Logical Memory I

This is a test of immediate verbal declarative memory from the Wechsler Memory Scale-III^UK ^[54]. It involves the immediate recall of a story with 25 elements which is read aloud. Two stories are administered, with recall after each one. The second story is administered twice. Participants are informed that they will be asked about the stories again later.

##### Verbal fluency

This task is said to assess executive function [55]. The participant is asked to name as many words as possible beginning with the letters C, F, and L, and is given one minute for each letter. Proper names are not allowed and repeated words are scored only once.

##### Prior cognitive ability

Two tests that estimate prior cognitive ability were administered. Both the National Adult Reading Test [56] and the Wechsler Test of Adult Reading [57] require the participant to pronounce 50 irregular words.

##### Digit symbol coding

This subtest from the Wechsler Adult Intelligence Scale-III^UK ^[58] was used to assess speed of information processing. The participant enters a symbol according to a given number-symbol code, completing as many as possible in two minutes.

##### Backward digit span

This is a test of working memory from the WMS-III^UK^. Increasingly-long strings of digits are read at a given rate by the tester, and the participant repeats them backwards.

##### Simple and four-choice reaction time

Mean and standard deviation of simple reaction time and four-choice reaction time were used to assess speed and variability of simple information processing. The tasks were administered using a stand-alone shallow rectangular box constructed for the UK Health and Lifestyle Survey [59]. This was described in detail and illustrated previously [60]. On the top face there is a high-contrast liquid crystal display (LCD) screen. There are five response keys arranged in a shallow arc and numbered, from left to right, 1, 2, 0, 3, 4. In the simple reaction time test there are 8 practice trials and 20 test trials. The participant rests the second finger of the preferred hand on the 0 key. After a zero appears on the LCD screen the participant presses the key as fast as possible. The mean and standard deviation of the 20 simple reaction time trials are calculated. The four-choice reaction time test has 8 practice trials and 40 test trials. The participant rests the second and third fingers of the left and right hands on, respectively, the keys marked 1, 2, 3, 4. After a number appears on the LCD screen the participant presses the appropriate key as quickly as possible. Each of the four numbers appears ten times, in a randomised order. Separate means and standard deviations are computed for correct and incorrect trials. For both the simple and four-choice reaction time trials there was a variable interval of between 1 and 3 seconds between the participant's response and onset of the next stimulus.

##### Logical Memory II

To test delayed verbal declarative memory, participants were asked to recall as much as possible from the two stories read to them in the Logical Memory I test.

##### Block design

This subtest from the WAIS-III^UK ^was used to assess constructional ability. The block design subtest requires participants to use blocks to make specific designs. They are given a maximum of two minutes to complete each design.

##### Verbal paired associates

This is a test of verbal learning and memory from the WMS-III^UK^. The participant is read a list of pairs which include words having no obvious connection. They are then given the first of the pair and asked to recall the other word. There are eight word pairs, and the same list, in different orders, is administered four times.

##### Spatial span

This is a test of non-verbal, spatial learning and memory from the WMS-III^UK^. The participant watches while the tester touches the top of a number of blocks in a spatial array. The task is to touch the same blocks in the same order. More difficult items involve larger numbers of blocks being touched. The task is repeated with the participant being required to touch the blocks in the reverse order.

##### Symbol search

This subtest from the WAIS-III^UK ^was used to assess speed of information processing. Each item requires the participant to examine a row of symbols to see if it contains one of a pair of target symbols. They indicate yes or no as quickly as possible and they complete as many items as possible in the allotted time.

##### Letter-number sequencing

This subtest from the WAIS-III^UK ^was used to assess working memory. Participants listen while the tester reads mixed, and increasingly long, strings of numbers and letters. They repeat them to the tester, with the numbers first, in numerical order, and then the letters in alphabetical order.

##### Matrix reasoning

This subtest from the WAIS-III^UK ^was used to assess non-verbal reasoning. In each item the participants examine a pattern arrayed in matrix with a piece missing. The elements of the matrix are arrayed according to rules. The task is to work out the rules, apply them to find out what the missing piece should look like, and choose the correct piece from the answer options.

##### Verbal paired associates delay

Without the pairs being read out again, participants are given the first word from each pair and asked to recall the second one. After this test, the physical examination was conducted and the blood samples were taken (see below).

##### Inspection time

This is a two-alternative, forced choice, backward masking, visual discrimination task. It was used to assess speed of elementary visual processing. The inspection time task was replicated as closely as possible from the one described in a previous study [61], but with a longer instruction period and more practice trials. The participants were required to make a simple visual discrimination: to indicate, with no pressure on response time, which of two parallel, vertical lines of markedly different lengths was longer. The inspection time test was constructed, run, and analyzed using E-Prime (Psychology Software Tools, Pittsburgh, PA). The stimulus lines were 5 cm for the longer line and 2.5 cm for the shorter line. They were joined at the top with a 2.5 cm crossbar. The lines were about 1.6 mm wide. The backward mask was constructed of a jumble of vertical lines 1.6 mm wide that overwrote the vertical lines in the stimulus. Participants were seated comfortably, with their eyes about 75 cm from a computer screen, though this was not fixed. A small fixation cross preceded the stimulus. This cue lasted 500 ms, and there was a blank interval of 800 ms between cue offset and stimulus onset. Ten trials were presented at each of 15 durations (rounded to the nearest millisecond): 6, 12, 19, 25, 31, 37, 44, 50, 62, 75, 87, 100, 125, 150, and 200. The backward mask lasted 500 ms. All stimuli were presented on a computer screen running at a vertical refresh rate of 160 Hz. On each trial, after mask offset, participants indicated the position of the longer line by pressing 1 (with the index finger of the right hand for 'left') or 2 (with the middle finger of the right hand for 'right') on the number pad of a computer keyboard. The correctness of each response was noted.

##### Moray House Test No. 12

This was taken at about age 11 on June 4^th ^in the Scottish Mental Survey of 1947 [51]. It was re-administered when participants were seen again at about age 70, using the same instructions and 45-minute time limit. Only two small changes were made to items whose content had become archaic [10]. The test is often referred to as a 'verbal' or 'verbal reasoning' test. However, the test has items of a variety of types: following directions (14 items), same-opposites (11), word classification (10), analogies (8), practical items (6), reasoning (5), proverbs (4), arithmetic (4), spatial items (4), mixed sentences (3), cypher decoding (2), and other items (4). A score of 76 was the maximum possible in the Moray House Test (MHT). With the collaboration of the SCRE, records from the original SMS1947 ledgers (held at the SCRE Centre's office in the University of Glasgow) containing MHT scores were checked to ensure a complete and accurate electronic index of individuals who sat the MHT. The SMS1947 database was used to obtain the MHT scores for all participants who attended the LBC1936 study and gave permission.

#### Physical examination and interview

This recorded: height; weight; corrected and uncorrected visual acuity in the right and left eyes using a Snellen chart; the time to walk 6 m; ability to stand from sitting; demi-span; head circumference; the Townsend's 9-item scale to assess activities of daily living [62]; sitting and standing systolic and diastolic blood pressure using an Omron 705IT monitor; lung function assessing peak expiratory flow rate, forced expiratory volume in 1 s, and forced vital capacity (each the best of three), using a Micro Medical Spirometer; grip strength in the right and left hand using a North Coast Hydraulic Hand Dynamometer (JAMAR); and date of the menopause for women.

#### Blood samples

Blood samples were taken for: DNA extraction from white blood cells; red and white blood cells and plasma, and for peripheral blood leucocyte immortalisation; full blood count, red cell folate and glycated haemoglobin (HbA1c); basic blood biochemistry, creatinine, lipid profile, thyroxine (T3, T4), thyroid stimulating hormone, albumin; a coagulation screen; and C-reactive protein.

### LBC1936 Study Questionnaire

Participants were asked to fill in questionnaires after their clinic visit and return these to the study team using a stamped addressed envelope provided. Participants were sent a reminder if the questionnaires had not been received within 6 weeks of their clinic visit. As the questionnaires were received into the office they were checked for missing or ambiguous answers (e.g. marking two responses instead of one) and corrections sent to the participant. These were generally promptly returned. The LBC1936 Study Questionnaire is a 20-page questionnaire booklet arranged in 5 sections, covering various aspects of the participants' lives.

#### LBC1936 Study Questionnaire Section 1

This was entitled 'Your Family' and asked questions relating to the participants' sociodemographic background, including parents' jobs, education & morbidity, the birthplace of their parents and grandparents and details relating to their children's year of birth, sex, education & occupation.

#### LBC1936 Study Questionnaire Section 2

This related to activities and consisted firstly of two questions relating to their general level of physical activity: this was assessed on a 6-point scale from movement associated with necessary (household) chores to keep-fit/heavy exercise or competitive sport, an item based on previous research [63]. Participants were then asked to indicate how many days in an average month they took part (for more than 20 minutes at a time) in any vigorous sport or physical exercise. The second part of this section consisted of a measure of their participation in a selection of 15 intellectual and social activities, drawn from those most commonly used in previous work in the field [64,65]. These included, for example, visits to the library, watching television, visits to friends or family or trips to the theatre or sporting events, and the frequency of participation was measured on a 5-point scale (from every day to less than once a year/never). Additional spaces were provided for participants to note activities in which they took part but that weren't listed in the table. Participants were then asked to provide details of lifelong learning experiences they had had in three broad categories: compulsory training related to their working lives, voluntary learning activities since school, and any recent learning activities.

#### LBC1936 Study Questionnaire Section 3

This incorporated two well-validated personality inventories: the IPIP and the NEO-FFI. The IPIP Big-Five Factor markers is a 50 or 100-item inventory that can be freely downloaded from the internet for use in research [66]. The current study made use of the 50-item version consisting of 10 items for each of the Big-Five personality factors: Extraversion (E), Agreeableness (A), Conscientiousness (C), Emotional Stability (ES) and Intellect (I). For each of the items, which are in sentence fragment form (e.g., "Am the life of the party"), "I" was added at the beginning so that the items would be easier to read, and more closely match the other inventories used. Participants were requested to read each of the 50 items and then rate how well they believed it described them on a 5-point scale (very inaccurate to very accurate). The NEO Five Factor Inventory [67] is a 60-item inventory consisting of 12 items each for the 5 factors: Neuroticism (N), Extraversion (E), Openness (O), Agreeableness (A) and Conscientiousness (C). Participants were asked to mark each item on a 5-point scale (strongly disagree to strongly agree) according to how well it described them.

#### LBC1936 Study Questionnaire Section 4

This aimed to measure participants' satisfaction with their lives. The WHOQOL-100 Quality of Life Assessment was developed by a group of WHO collaborators in 15 international field centres simultaneously to produce an assessment of an individual's quality of life beyond their physical health that would be applicable cross-culturally. The WHOQOL-BREF was developed using data from this as an abbreviated version for use in, for example, large epidemiological studies where quality of life is only one variable of interest. It produces scores for four domains related to quality of life: physical health, psychological, social relationships and environment as well as one facet on overall quality of life and general health, and has been shown to demonstrate good validity, consistency and reliability [68]. The WHOQOL-BREF was used in the LBC1936 study for its brevity and conciseness and all but one of the 26 questions was utilised; the question regarding participants' sex lives was not deemed appropriate for this age group.

#### LBC1936 Study Questionnaire Section 5

This section ('Support from others') assessed the social support and network characteristics in the LBC1936. The structural characteristics of the participant's social network were first assessed; they were asked about their current living arrangement and how long they had lived in this situation, and the number of 'close' friends and relatives they could depend on. The latter item was adapted from previous research [69-71]. The presence or absence of a confidant (providing emotional support), and the presence or absence of someone to provide practical support/assistance was then assessed, with the adequacy of the latter item also recorded. These 3 items were adapted from a previous study [69]. Participants were then asked to indicate whether they had had social interaction with a family member and a friend in the past 2 weeks, how often they themselves felt lonely at the present time and whether or not they felt they had someone they could talk to when they had problems. The perceived availability of, and satisfaction with, six specified types of social support received were then assessed. These items were adapted from the Social Support Questionnaire-Short Form [72] based on item wordings used in previous research [68]. For each item (for example, "How often were there people you could really count on to be dependable when you needed help?"), participants were required to report how frequently people were available to provide the specified type of support on a 5-point scale (from all of the time to none of the time). Participants then stated how satisfied they were with this level of support on a 6-point scale (from very satisfied to very dissatisfied).

### Food Frequency Questionnaire

Participants were given a diet questionnaire to complete and return alongside the LBC1936 Study Questionnaire. The Scottish Collaborative Group Food Frequency Questionnaire, version 7.0, was used [73]. This consists of a list of 175 items of food or drink presented in 23 sections. Participants were asked to indicate how often they consumed a pre-determined measure of each of these items over the last 2–3 months, ranging from 'rarely or never' through to '7+ a day'. Supplementary questions were included to clarify consumption of various items including bread, sugar, spreads and oils, vitamins, minerals and food supplements, and foods not included elsewhere in the questionnaire. The Food Frequency Questionnaires were sent to the University of Aberdeen for nutrient extraction, and the data then added into the overall analysis.

### Data checking

#### Clinic visit data

On completion of data entry, a sample of 100 of the complete LBC1936 clinic visit packs (containing demographic, cognitive testing and medical data information) were checked in their entirety against their respective entries in the SPSS database to ensure accuracy of data entry. We found insufficient errors in the sample of 100 to warrant a complete data entry check. All have been kept in a data log book. There were errors of entry in 30 out of 22,400 cells (0.13%; 100 subjects each with 224 columns of data). Errors were mostly small, numerical errors in cognitive test scores, blood results and medical data (e.g. height and lung function). In several cases the error occurred after a decimal point. There were no systematic errors. All errors and discrepancies found were noted in a log, checked and amended on the SPSS database.

#### Questionnaire data

A sample of 100 LBC1936 questionnaires (containing family history, personality and social items) were checked against our records to ensure accuracy of data entry. We found insufficient errors (about 45) in the sample of 100 questionnaire entries to warrant a complete data entry check. There were 41 errors of entry in 34,500 cells (0.12%; 100 subjects each with 345 columns of data), and 7 cells with missing information. The main data entry errors were found in cells containing data from the IPIP and NEO sections of the questionnaires, where individual item responses coded as 1, 2, 3, 4, or 5 were sometimes discrepant by one point, e.g. a 4 instead of a 5. There were no systematic errors. All errors and discrepancies found were checked and amended on the SPSS database, and entered into a data log book.

Once the SPSS databases had been checked and updated, frequencies were run on all numerical input to ensure that all results were within the appropriate scales, and any errors were checked against the original, handwritten datasheets and corrected as necessary.

#### DNA quality control

Monthly reports generated by the Wellcome Trust Clinical Research Facility Genetics Core at the Western General Hospital Edinburgh, containing up-to-date information for blood work performed on LBC1936 samples, including LBC number, name, date of birth, and date of attendance, were checked against records to ensure consistency and discrepancies were verified and forwarded to the Genetics Core for amendment. The results of DNA sex-typing analysis, performed by the Genetics Core for all samples, were cross-matched to the LBC1936 databases to confirm that correct labelling of samples had taken place. This revealed several errors, four of which were caused by mixing-up of pairs of blood tubes at the extraction or labelling stage. These were rectified by re-analysis or the provision of a second sample from those participants.

## Discussion

The present protocol is being reported at the point where the data have been collected and checked. In discussing this protocol we refer back to the nine research objectives which were stated in the application for funds to Research Into Ageing. Research objectives 1 to 4 have been achieved by the completion of data collection according to this protocol. The LBC1936 data will now be used to test the hypotheses stated in research objectives 5 to 7. Research objective 8 has been achieved by obtaining further support, from Help the Aged's Disconnected Mind project, to continue examining the LBC1936 in subsequent waves. Research objective 9 has been achieved by there now being a resource that may be used by other researchers.

A particular strength of the study is the availability of well-validated mental ability test scores at age 11, and the fact that these scores can be compared with the entire population of Scotland born in 1936 and attending schools in Scotland in June 1947. The important domains of cognitive functioning have been examined: reasoning, memory, speed of information processing, and executive function. There are especially rich data on speed of information processing and memory. There are rich data on social and biological factors as possible contributors to individual differences in lifetime cognitive ageing.

## List of abbreviations used

CHI: Community Health Index

IPIP: International Personality Item Pool

LBC1936: Lothian Birth Cohort 1936

LCD: liquid crystal display

MHT: Moray House Test

SCRE: Scottish Council for Research in Education.

SMS1947: Scottish Mental Survey 1947

WAIS-III^UK^: Wechsler Adult Intelligence Scale-III^UK^

WHOQOL: World Health Organisation Quality of Life

WMS-III^UK^: Wechsler Memory Scale-III^UK^

## Competing interests

The author(s) declare that they have no competing interests.

## Authors' contributions

The principal investigator on the Lothian Birth Cohort 1936 grant was IJD, and JMS, VW, HC, LJW, DJP and PV were co-investigators. The investigators designed the study, with IJD and JMS taking principal responsibility for compiling the psychological and medical phenotypes, respectively. MDT and IJD revised the protocol for testing subjects. AJG contributed to the LBC1936 Study Questionnaire design. MDT, AJG, JC, CB and CC collected, entered and checked the data. IJD drafted the article, with sections contributed by AG, JC and CB. All authors read and contributed to revising the article.

## Pre-publication history

The pre-publication history for this paper can be accessed here:


